# Immune response to vaccination in people with psychotic disorders relative to healthy controls: prospective study of SARS-CoV-2 vaccination

**DOI:** 10.1192/bjo.2024.10

**Published:** 2024-02-16

**Authors:** Oisín O'Brien, Atheeshaan Arumuham, Yuya Mizuno, Luke Baxter, Maria Lobo, Sita Parmar, Stephen Jolles, Oliver D. Howes

**Affiliations:** Department of Psychosis Studies, Institute of Psychiatry, Psychology and Neuroscience, King's College London, UK; South London and Maudsley NHS Foundation Trust, London, UK; and Institute of Clinical Sciences, Faculty of Medicine, Imperial College London, UK; Department of Psychosis Studies, Institute of Psychiatry, Psychology and Neuroscience, King's College London, UK; South London and Maudsley NHS Foundation Trust, London, UK; and Department of Neuropsychiatry, Keio University School of Medicine, Tokyo, Japan; South London and Maudsley NHS Foundation Trust, London, UK; Immunodeficiency Centre for Wales, University Hospital of Wales, Cardiff, UK

**Keywords:** Schizophrenia, SARS-CoV-2, immune response, vaccine, health policy

## Abstract

This prospective study examines the immune response to SARS-CoV-2 vaccination in patients with psychotic disorders compared with healthy volunteers. Participants were recruited naturalistically as part of the UK's COVID-19 vaccination programme. Prior to receiving their first COVID-19 vaccine, blood samples were provided by participants to examine anti-SARS-CoV-2 immunoglobulins (IgG) at baseline, followed by a repeat assay 1 month after receiving their first vaccine to assess vaccine response. The increase of IgG levels from baseline to 1 month post-vaccination was significantly lower in patients compared with controls, supporting evidence of impaired vaccine response in people with psychotic disorders. When excluding patients treated with clozapine from the analysis, this difference was no longer significant, suggesting that effects may be particularly marked in people taking clozapine.

Schizophrenia and related psychotic disorders are associated with higher COVID-19 mortality rates than in age- and sex-matched controls,^1^ even after adjusting for comorbidities.^2^ Although illness behaviour and low vaccine uptake contribute,^3^ several lines of evidence indicate that immune dysfunction may also play an important role in these poor outcomes following COVID-19 infection. In particular, schizophrenia has been associated with genetic loci implicated in immune function^4^ and higher serum levels of pro-inflammatory cytokines relative to controls.^5^ Vaccine response has also been reported to be lower in people with schizophrenia relative to healthy controls.^6^ Clozapine may be a particular risk factor for this as people treated with clozapine present with lower immunoglobulin (IgG) levels, similar to levels seen in primary immunodeficiency, and that resolve on cessation of clozapine.^7^ Finally, people with a psychotic disorder have an increased risk of breakthrough COVID-19 infection despite vaccination, relative to healthy controls.^8^ These lines of evidence suggest that there could be an impaired immune response to COVID-19 vaccination in psychotic disorders. However, to our knowledge this has not been tested. In view of this, we aimed to test the hypothesis that the immune response to COVID-19 vaccination is lower in people with schizophrenia relative to controls.

## Method

The study was approved by the London-Surrey NHS research ethics committee (ref: 20/HRA/1987) and complies with the Helsinki Declaration of 1975, as revised in 2008. All participants gave written informed consent to participate. Between February 2021 and February 2022, we recruited patients aged ≥18 years from mental health teams in London (UK) with a diagnosis of a psychotic disorder meeting ICD-10 criteria (codes F20–F29) and healthy controls from the same geographical area with no history of psychotic disorder (confirmed using the the Structured Clinical Interview for DSM-5 – Clinician Version (SCID-5-CV)). No participant had previously received a COVID-19 vaccine.

Participants received a baseline screening and clinical assessment, and provided a baseline blood sample 0–12 days prior to their first COVID-19 vaccination. The brand of vaccine offered was either AstraZeneca ChAdOx1-S, Pfizer BioNTech BNT162b2 or Moderna mRNA-1273, depending on availability. A follow-up blood sample was taken 1 month after vaccination.

The EUROIMMUN Anti-SARS-CoV-2 assay was used to measure serum levels of immunoglobulins specific to the SARS-CoV-2 spike protein. This enzyme-linked immunosorbent assay (ELISA) provides semi-quantitative *in vitro* determination of human anti-SARS-CoV-2 spike protein IgG antibodies. Intra-assay and inter-assay variations have been estimated as <7% and <5% respectively; sensitivity and specificity have been reported as 91.39% and 98.56% respectively.^9^ Results are expressed as the ratio of the extinction of the sample over the extinction of the calibrator, and are interpreted in terms of IgG response to vaccine as follows: <0.8 denotes negative; ≥0.8 to <1.1 borderline/indeterminate; ≥1.1 positive.^9^

SPSS version 22 for Mac OS was used for all statistical analyses and the significance level was set to *P* < 0.05. Our primary outcome measure is vaccine response, calculated as the change in SARS-CoV-2 spike protein IgG level:

log_e_[IgG level 1 month post-vaccination] – log_e_[IgG level pre-vaccination]

We used independent *t*-tests to test our main hypothesis that there would be a lower response in patients compared with controls, and to compare continuous clinical and demographic variables at baseline. A two-tailed *P*-value <0.05 was considered significant. One-way analysis of covariance (ANCOVA) was performed to control for covariates of interest, namely age and sex.

## Results

In total, 24 patients and 33 controls participated. Clinical and demographic variables and vaccine response are summarised in Table 1. Our cohorts were matched for age (*t*_(55)_ = −1.91, *P* = 0.065), although our patient group consisted of more males than our control group (Fisher's exact test, *P* < 0.001).

**Table 1:** Clinical and demographic variables by group (healthy controls versus people with psychotic disorders)

	Controls (*n* = 33)	Patients (*n* = 24)	Statistical comparison
Age, years: mean (s.d.)	28.8 (7.5)	34.4 (12.9)	*t*_(55)_ = −1.91; *P* = 0.065
Sex, male/female: *n*	8/25	18/6	Fisher's exact *P* < 0.001
ICD-10 diagnosis, *n*	n.a.	F20 schizophrenia: 18; F25 schizoaffective disorder: 1; F29 unspecified non-organic psychosis: 5	n.a.
Illness duration, years: mean (s.d.)	n.a.	10.46 (10.46)	n.a.
Comorbid conditions, *n*	Hypertension: 0	Hypertension: 1	n.a.
	T2DM: 0	T2DM: 1	
	COPD: 0	COPD: 1	
	Asthma: 1	Asthma: 1	
Receiving antipsychotic, *n*	n.a.	None: 1; aripiprazole: 7; clozapine: 8; olanzapine: 4; quetiapine: 1; risperidone: 3	n.a.
Daily chlorpromazine equivalent dose, mg: mean (s.d.)	n.a.	331.6 (144.7)	n.a.
First vaccine type received, AZ/Pfizer/Moderna: *n*	4/26/3	7/17/0	Fisher's exact *P* = 0.13
Anti-SARS-CoV-2 IgG antibodies at baseline, *n*: mean (s.d.)	0.72 (1.17)	1.25 (1.68)	*t*_(55)_ = −1.33; *P* = 0.19
Anti-SARS-CoV-2 IgG antibodies at 1-month follow-up, *n*: mean (s.d.)	4.67 (1.65)	4.19 (2.09)	*t*_(55)_ = 0.97; *P* = 0.34

n.a., not applicable; T2DM, type 2 diabetes mellitus; COPD, chronic obstructive pulmonary disease; AZ, AstraZeneca ChAdOx1-S; Pfizer, Pfizer BioNTech BNT162b2; Moderna, Moderna mRNA-1273; IgG, immunoglobulin.

IgG levels were not normally distributed and so were log(normal) transformed. The increase of IgG levels from baseline to 1 month post-vaccination was significantly lower in patients (+1.86, s.d. = 1.16) than in controls (+2.45, s.d. = 0.89; t_(55)_ = 2.2, *P* = 0.035). The group difference remained significant when sex was included as a covariate (F_(1,54)_ = 4.38, *P* = 0.04) and at a trend level significance with age as a covariate (F_(1,54)_ = 3.61, *P* = 0.06). Three patients and zero controls failed to achieve a ‘positive’ level (≥1.1) after first vaccination. All three patients who did not exhibit an adequate response to vaccination were taking clozapine. As a sensitivity analysis, we excluded patients on clozapine, and found there was no longer a significant difference between patients (+1.93, s.d. = 1.14) and controls (+2.45, s.d. = 0.89; t_(47)_ = 1.7, *P* = 0.09). We also ran a sensitivity analysis with sex-ratio-matched patients and controls (males = 8, females = 6 in each group) with computer generated randomly selected cases, and found that the significantly lower increase in IgG levels in the patient group remained (t_(26)_ = 2.99, *P* = 0.006).

## Discussion

We show that antibody response to COVID-19 vaccination was lower in patients with psychotic disorders than in healthy controls. Additionally, three clozapine-treated patients failed to seroconvert after one vaccine dose.

Our findings could suggest that an impaired immune response to the COVID-19 spike protein underlies prior evidence that people with psychotic disorders are at higher risk of mortality from SARS-CoV-2.^1,2^ To our knowledge, this is the first study to directly measure serological response to COVID-19 vaccination in people with psychotic disorders. We had the advantage of recruiting both patients and controls in the same time period and geographical location, when COVID-19 vaccines first became available to the general public, which allowed us to obtain baseline measurements shortly before first vaccination. Recruitment during this public health initiative was opportunistic, limited to a finite pool of people who remained vaccine-naive, and also targeted a patient cohort known to have lower vaccine uptake rates than the general population.^3^ A limitation of the assay used in this study was that it experienced saturation at high levels. Nevertheless, this is unlikely to explain our findings, as it would affect both groups similarly.

Sensitivity analyses indicate that age could contribute to the lower immune response in patients. This potential relationship with age has been reported in studies assessing the efficacy of COVID-19 vaccination across different age groups, with the immune effect of young people being vaccinated being more robust than in older people.^10^ Moreover, people with schizophrenia are noted to have accelerated aging, which may be further driving an impaired immune response.^11^

Our observation that three patients did not meet criteria for an adequate IgG response to vaccination indicates this may be a potentially clinically important effect; they were all taking clozapine. Clozapine is known to cause immunodeficiency and poor response to other vaccines,^7^ so these results suggest it may reduce the likelihood of seroconversion in some cases. However, these results should be interpreted cautiously as the sample is small and the study was not designed to compare treatment effects. Nevertheless, a review of literature indicates that patients treated with clozapine were not at higher risk of contracting COVID-19 or developing complications of illness than patients treated with non-clozapine antipsychotics.^12^ Further research is required to investigate the long-term efficacy of COVID-19 vaccines, as well elucidating whether alterations in immune response in people with schizophrenia is solely attributable to clozapine. Our findings extend prior evidence of impaired responses to other vaccines in people with psychotic disorders.^6^ Future studies should aim to carefully control for demographic variables (e.g. age, sex, ethnicity) and other factors that might influence vaccine response.

## Data Availability

The data that support the findings of this study are available from the corresponding author, O.D.H., on reasonable request.
